# Supplementary material for: Complexome profiling of the Chlamydomonas psb28 mutant reveals TEF5 as an early PSII assembly factor
Source: Plant Cell. 2025 Mar 17;37(6):koaf055. doi: 10.1093/plcell/koaf055 (PMC12164590; doi:10.1093/plcell/koaf055)
Supplement: koaf055_Supplementary_Data [file koaf055_supplementary_data.zip › psb28_interactive.zip!/plots/intensity/qualityPlots/peptides/ZEP1_Cre02.g082550.t1.2.html]

### Cre02.g082550.t1.2

Trivial name: ZEP1  
SeqCoverage [median]: 13.40  
Mass: 80.96 kDa  
  
 

| Sequence | MissedCleavage | Mass [Da] | wtIntensity | muIntensity | Score |
| --- | --- | --- | --- | --- | --- |
| AGNAAAVDVEGVLR | 0 | 1340.70 | 3076.74 | 3032.66 | 91.94 |
| ATPEEDVLRR | 1 | 1184.61 | 3728.47 | 2129.76 | 67.33 |
| AVSDKAGNAAAVDVEGVLR | 1 | 1840.96 | 637.80 | 907.62 | 54.97 |
| FPEFMNNDASIIR | 0 | 1552.73 | 366.34 | 618.81 | 83.86 |
| LTLQQILAK | 0 | 1026.64 | 3541.10 | 0.00 | 143.61 |
| QGFQVQVFER | 0 | 1236.63 | 347.45 | 0.00 | 111.61 |
| QLIGETK | 0 | 787.44 | 836.23 | 0.00 | 77.74 |
| RNDTTGNNEVTVQLEDGR | 1 | 2016.95 | 870.03 | 1234.65 | 87.16 |
| SDELSGQAYTTLMVGK | 0 | 1698.81 | 0.00 | 918.81 | 85.29 |
|  |  |  | 13404.2 | 8842.3 |  |
